# MicroRNA-133a acts as a tumour suppressor in breast cancer through targeting LASP1

**DOI:** 10.3892/or.2022.8330

**Published:** 2022-05-16

**Authors:** Yanmin Sui, Xiaolei Zhang, Honglan Yang, Wei Wei, Minglin Wang

Oncol Rep 39: 473–482, 2018; DOI: 10.3892/or.2017.6114

Following the publication of this paper, it was drawn to the Editors attention by a concerned reader that the western blotting assay data shown in Figs. 5B, 5E, 6C and 7A were strikingly similar to data appearing in different form in other articles by different authors. Owing to the fact that the contentious data in the above article had already been published elsewhere, or were already under consideration for publication, prior to its submission to *Oncology Reports*, the Editor has decided that this paper should be retracted from the Journal. The authors were asked for an explanation to account for these concerns, but the Editorial Office did not receive a reply. The Editor apologizes to the readership for any inconvenience caused.

